# New Laboratory Protocol to Determine the Oxidative Stress Profile of Human Nasal Epithelial Cells Using Flow Cytometry

**DOI:** 10.3390/jcm10061172

**Published:** 2021-03-11

**Authors:** Ana Reula, Daniel Pellicer, Silvia Castillo, María Magallón, Miguel Armengot, Guadalupe Herrera, José-Enrique O’Connor, Lucía Bañuls, María Mercedes Navarro-García, Amparo Escribano, Francisco Dasí

**Affiliations:** 1 Department of Physiology, School of Medicine, University of Valencia, Avda. Blasco Ibáñez, 17, 46010 Valencia, Spain; ana.reula@uv.es (A.R.); dpellicerroig@gmail.com (D.P.); mariamagallon94@gmail.com (M.M.); lucia.banyuls.soto@gmail.com (L.B.); 2 Rare Respiratory Diseases Research Group, IIS INCLIVA, Fundación Investigación Hospital Clínico Valencia, Avda. Menéndez y Pelayo, 4, 46010 Valencia, Spain; sccorullon@gmail.com (S.C.); mer_navarro2002@yahoo.es (M.M.N.-G.); aescribano@separ.es (A.E.); 3 Pediatrics Unit, Hospital Clínico Universitario Valencia, 46004 Valencia, Spain; 4 Department of Surgery, School of Medicine, University of Valencia, Avda. Blasco Ibáñez, 17, 46010 Valencia, Spain; miguel.armengot@uv.es; 5 ENT Unit, Hospital La Fe, 46026 Valencia, Spain; 6 Flow Cytometry Unit, IIS INCLIVA, Fundación Investigación Hospital Clínico Valencia, Avda. Menéndez y Pelayo, 4, 46010 Valencia, Spain; guadalupe.herrera@uv.es; 7 Department of Biochemistry, School of Medicine, University of Valencia, Avda. Blasco Ibáñez, 17, 46010 Valencia, Spain; jose.e.oconnor@uv.es; 8 Department of Pediatrics, Obstetrics and Gynecology, School of Medicine, University of Valencia, Avda. Blasco Ibáñez, 17, 46010 Valencia, Spain

**Keywords:** flow cytometry, rare respiratory diseases, nasal epithelium, oxidative stress, reactive oxygen species

## Abstract

Several studies have shown the importance of oxidative stress (OS) in respiratory disease pathogenesis. It has been reported that the nasal epithelium may act as a surrogate for the bronchial epithelium in several respiratory diseases involving OS. However, the sample yields obtained from nasal biopsies are modest, limiting the number of parameters that can be determined. Flow cytometry has been widely used to evaluate cellular OS profiles. It has the advantage that analyses can be performed using a small amount of sample. Therefore, we aimed to set up a new method based on flow cytometry to assess the oxidative profile of human nasal epithelial cells which could be used in research on respiratory diseases. Levels of total nitric oxide, superoxide anion, peroxynitrite, and intracellular peroxides were measured. Reduced thiol levels, such as antioxidant-reduced glutathione and oxidative damaged lipids and proteins, were also analysed. The intracellular calcium levels, plasma membrane potential, apoptosis, and percentage of live cells were also studied. Finally, a strategy to evaluate the mitochondrial function, including mitochondrial hydrogen peroxide, superoxide anion, mitochondrial mass, and membrane potential, was set up. Using small amounts of sample and a non-invasive sampling technique, the described method enables the measurement of a comprehensive set of OS parameters in nasal epithelial cells, which could be useful in research on respiratory diseases.

## 1. Introduction

Free radicals are molecules with at least one unpaired electron in their outer layer. The need to acquire an electron to achieve electrochemical stability drives reactions of free radicals with other biomolecules. Free radicals react with DNA, lipids, and proteins, producing oxidation and a loss of activity in these biomolecules [1]. As a by-product of cellular aerobic metabolism, various reactive species are generated, including reactive oxygen species (ROS), such as superoxide (O_2_^−^) or hydrogen peroxide (H_2_O_2_), and reactive nitrogen species (RNS), such as nitric oxide (NO) or peroxynitrite (ONOO^−^) [2,3]. The primary intracellular sources of ROS and RNS are mitochondria, lysosomes, peroxisomes, nuclear and cytoplasmic membranes, and the endoplasmic reticulum [4]. ROS and RNS are also generated by external factors, such as tobacco or environmental pollution [5]. Eukaryotic cells possess defence mechanisms to avoid biomolecular oxidative damage, with vitamin C, vitamin E, and glutathione all relevant examples of simple defence mechanisms [6]. Cells also have complex enzymatic systems, such as superoxide dismutase (SOD), catalase (CAT), glutathione peroxidase (GPx), and glutathione reductase (GR), which evolved to reduce ROS levels [7,8]. Under physiological conditions, basal ROS and RNS levels are necessary for cells’ function by acting as regulatory and signalling molecules. Thus, cells need these species to maintain the cellular reduction-oxidation (REDOX) balance [9].

Oxidative stress (OS) is produced when there is an imbalance toward pro-oxidation between pro-oxidant and antioxidant systems. When defensive mechanisms cannot prevent ROS and RNS accumulation, signalling pathways are activated and gene expression and protein synthesis changes occur [9]. In response to DNA oxidative damage, cells react by repairing the damage, activating different cell cycle checkpoints, or inducing apoptosis [10]. These conditions are linked to numerous pathological processes, such as cancer, chronic inflammation, ageing, neurodegenerative and cardiovascular disease, and asthma, among others [11,12].

Nasal epithelial cells represent the first line of defence against various environmental factors. These cells clean, humidify, and warm inhaled air and produce mucus, which attaches to particles transported by cilia present on some cells to the digestive tract, where they are eliminated. Nasal epithelial cells are not only a physical barrier but were also shown to respond by producing inflammatory mediators that can affect the local immune response [13]. These cells are suitable in vitro models for the study of novel defence mechanisms [14]. Thus, the nasal epithelium may act as a surrogate for the bronchial epithelium in asthma studies [15].

On the other hand, numerous studies have shown the importance of OS as a factor involved in the pathogenesis of several diseases of the respiratory system, such as Chronic Obstructive Pulmonary Disease (COPD) [16], asthma [17], alpha-1 antitrypsin deficiency (AATD) [18,19,20,21] and primary ciliary dyskinesia (PCD) [22]. Therefore, it is essential to know the underlying mechanisms by which OS directs pathogenesis in these diseases to develop more effective therapies. There are currently no methods based on flow cytometry that allow the analysis of different OS parameters in nasal epithelial cells. Therefore, given the importance of these cells in different respiratory pathologies and the important role of OS in their development, we present a new method based on flow cytometry to assess the oxidative profile of human nasal epithelial cells, which could be very useful in the future research of several respiratory diseases.

## 2. Experimental Section

### 2.1. Biological Samples

Nasal epithelial cells were obtained from six healthy donors (3 males and 3 females; ages (mean ± Standard Deviation) 27 ± 1.5 and 28 ± 2.3, respectively) at the Hospital Clínico Universitario de Valencia (HCUV) and Hospital General Universitario de Valencia (HGUV) (Valencia, Spain). A cytology brush (Covaca SA CE2005, Madrid, Spain) was inserted into the patient’s nostril, and the nasal epithelium of the middle meatus was gently brushed, yielding strips of ciliated nasal epithelium. Samples were transported using Medium199 supplemented with Hanks’ salts, l-glutamine, 25 mM HEPES, and 1% penicillin/streptomycin. Before labelling, samples were filtered using 50 µm CellTrics^®^ filters (Sysmex; Sant Just Desvern; Barcelona, Spain) (25004-0042-2317 Sysmex) to isolate cells from cell debris and aggregates. Participants were healthy, non-smoking individuals without respiratory bronchial disease, local or systemic disease, allergies, rhinosinusitis, or upper respiratory tract infection for at least one month before sampling. The study protocol was approved by the Ethics Committees of the HCUV and HGUV and followed the ethical guidelines of the 1975 Declaration of Helsinki [23]. All the participating individuals gave their written informed consent. Samples were analysed in the Cytometry Service of the Unidad Central de Investigación Medicina (UCIM) of Instituto de Investigación Sanitaria INCLIVA.

### 2.2. Reagents

The probes used in this study are detailed, including global distributors, in the Supplementary Materials (Reagents). Flow cytometry methods, including reagent concentrations; storage temperature; species studied; incubation protocols in basal tubes; and positive controls, cytometers, and lasers used for each measurement are summarized in Table 1 and Table 2. Additionally, a step-by-step protocol is available in the Supplementary Materials section.

### 2.3. Determination of Dead and Live Cells

Dead cells may compromise flow cytometric data analysis, especially when studying physiological conditions, such as OS. As cellular viability is usually determined by measuring cells’ capacity to exclude vital dyes, we excluded dead cells from our analysis by adding a DNA-binding dye. 4′,6-diamidino-2-phenylindole (DAPI) was added into tubes with dihydroethidium (HE), 2’-7’dichlorofluorescin diacetate (DCFH), Dihydrorhodamine 123 (DHR1,2,3), Diaminofluorescein-FM diacetate (DAF-FM DA), 5-chloromethylfluorescein diacetate (CMFDA), Tetramethylrhodamine, methyl ester (TMRM), MitoTracker Green, MitoSOX, MitoPY 1, FLUO-4, BODIPY 665 (B665), and FTC, whereas propidium iodide (PI) was used with Bis(1,3-dibutylbarbituric acid)trimethine oxonol (DIBAC). PI is widely used with Annexin V to determine if cells are viable, apoptotic, or necrotic through differences in plasma membrane integrity and permeability. PI does not stain live or early apoptotic cells due to the presence of an intact plasma membrane, but in late apoptotic and necrotic cells the integrity of the plasma and nuclear membranes decreases, allowing PI to cross the cell membrane, intercalate into nucleic acids, and display red fluorescence [24,25].

### 2.4. Reactive Oxygen Species and Reactive Nitrogen Species Assessment

Superoxide anion (O_2_^−^) is detected by HE, a fluorescent probe selectively oxidised and hydroxylated by O_2_^−^ to 2-OH-ethidium, emitting fluorescence when bound to DNA [26,27]. Hydrogen peroxide (H_2_O_2_) detection was based on DCFH oxidation, which generally exhibits a low basal fluorescence, but is converted to highly fluorescent DCF when oxidised by H_2_O_2_ in the presence of peroxidase [27,28]. Peroxynitrite (ONOO^−^) production was assessed using DHR1,2,3, an uncharged, nonfluorescent ROS indicator that passively diffuses across membranes where it is oxidised to cationic rhodamine 123, exhibiting green fluorescence [29,30]. Nitric oxide (NO) production was assessed using DAF-FM DA, an otherwise nonfluorescent probe that forms a fluorescent benzotriazole when it reacts with NO, thereby acting as a specific NO detector [31,32]. Intracellular thiol-reduced status, including reduced glutathione (GSH), was measured using CMFDA [33,34]. For each measurement, cells were incubated with the appropriate probe in individual tubes—i.e., HE (2.5 ug/mL), DCFH (2.5 μg/mL), DHR1,2,3 (100 μM), DAF-FM DA (1 μM), or CMFDA (25 nM)—for 20 min at 37 °C. Fluorescence was measured by flow cytometry with the appropriate settings.

### 2.5. Plasmatic Membrane Potential Assessment

Plasma membrane potential was evaluated using DIBAC, a potential-sensitive fluorescent probe that can enter depolarised cells and bind to intracellular proteins or membranes. Increased depolarisation results in an additional influx in the anionic dye and increases fluorescence. Conversely, hyperpolarisation results in a decrease in fluorescence. DIBAC dyes are excluded from mitochondria because of their negative charge, making them suitable for measuring plasma membrane potentials [35,36]. Cells were incubated with DIBAC (1.2 μM) for 20 min at 37 °C, and fluorescence was measured by flow cytometry with the appropriate settings.

### 2.6. Mitochondrial Assessment

Mitochondrial membrane potential (Ψ_m_) was analysed using TMRM, a fluorescent probe that accumulates inside mitochondria directly proportional to their membrane potential [37,38]. Mitochondrial mass was determined using MitoTracker Green, a fluorescent dye that locates mitochondria independently to Ψ_m_ [39,40]. Mitochondrial O_2_^−^ (mtO_2_^−^) was measured using MitoSOX, which enters live cells, specifically the mitochondria, where it is rapidly oxidised by O_2_^−^ [30,41]. Mitochondrial H_2_O_2_ (mtH_2_O_2_) was measured using mitoPY 1, a cell-permeable fluorescent probe that selectively tracks the mitochondria of living cells by selectively binding to mtH_2_O_2_ [30,42]. For each measurement, cells were incubated with the appropriate probe in individual tubes—i.e., TMRM (600 nM), MitoTracker Green (78 nM), MitoSOX (640 nM), or MitoPY 1 (4 μM)—for 20 min at 37 °C. Fluorescence was measured by flow cytometry with the appropriate settings.

### 2.7. Intracellular Calcium Assessment

Intracellular calcium (Ca^2+^) was measured using FLUO-4, a fluorogenic probe that detects intracellular Ca^2+^ [43,44]. Cells were incubated with FLUO-4 (0.5 μM) for 20 min at 37 °C, and fluorescence was measured by flow cytometry with the appropriate settings.

### 2.8. Oxidative Damage to Biomolecules

Lipid peroxidation was detected using the lipophilic probe BODIPY 665/676 dye, which exhibits a change in fluorescence emission after interaction with peroxyl radicals [45,46]. Cells were incubated with B665 (800 nM) for 30 min at 37 °C, and fluorescence was measured by flow cytometry with the appropriate settings.

Protein oxidation (carbonylation) levels were measured using FTC, a molecule that emits green fluorescence when it interacts with carbonyl groups of proteins [26,47]. Cells were incubated with FTC (800 nM) for 20 min at 37 °C, and fluorescence was measured by flow cytometry with the appropriate settings.

### 2.9. Apoptosis Assay

Apoptosis status was determined using Annexin V, a 35–36 kDa Ca^2+^-dependent phospholipid-binding protein with a high affinity for phosphatidylserine (PS), which binds to PS on exposed apoptotic cell surfaces [48]. Cells were incubated in the dark for 15 min at room temperature with Annexin V, PI, and Annexin V-binding buffer (previously diluted to 1/10 in PBS). After incubation, 300 μL of the 1/10 Annexin V-binding buffer was added to the dilution. Samples were analysed by flow cytometry with the appropriate settings.

### 2.10. Positive Control Incubations

As controls, appropriate fluorochromes were added to each tube after previous incubation with their respective inducers. An H_2_O_2_ generator, t-BHP (100 μM) [49,50], was used for the B665, DCFH, MitoPY 1, and DIBAC tubes. PB (2.24 μg/mL), an O_2_^−^ generator [50,51], was used for HE and MitoSOX tubes. DEM (20 mM), which produces GSH depletion [33,50], was used for CMFDA. Menadione (1 mM) was used for FTC to increase the carbonylated protein levels [50,52], and FCCP (52 μM), a mitochondrial uncoupler that decreases Ψ_m_ [50,53], was used for TMRM. Each tube was incubated in the dark for 15 min (except menadione, which was incubated for an hour, and DEM, which was incubated for 90 min) at 37 °C with their respective inducers. Next, each tube was incubated in the dark for 30 min at 37 °C. Samples were run on the flow cytometer with the appropriate settings.

A kinetic strategy was designed for the real-time follow-up of intracellular NO, ONOO^−^, and Ca^2+^ generation. Cells were previously incubated with their respective fluorochromes—i.e., DAF (1 μM), DHR1,2,3 (100 μM), or FLUO-4 (0.5 μM), and DAPI. Each tube was incubated in the dark for 20 min at 37 °C. Afterwards, the acquisition process was paused to add the inducers. In the NO-positive control, two doses of NOR-1 (16 μg/mL), a nitric oxide generator [35], were added, and the acquisition process was continued until 300 s. In the ONOO^−^-positive control, PB (2.24 μg/mL) and NOR-1 (16 μg/mL) were added, and the acquisition was continued until 200 s. In the Ca^2+^-positive control, the ionophore ionomycin (50 μM) was added to stimulate Ca^2+^ influx, and acquisition was continued until 600 s.

### 2.11. Cytometer Settings and Data Analysis

All flow cytometry assays were carried out using the FACSVerse cytometer (BD Biosciences, San Jose, CA, USA), except the lipid oxidised/lipid reduced ratio, which was carried out using the FacsAria III cytometer (BD Biosciences), and kinetic analyses of NO, ONOO, and Ca^2+^ generation, which were carried out using the LSR Fortessa X-20 (BD Biosciences) (Table 1).

Blue (488 nm), violet (405 nm), and red (635 nm) lasers were used. The fluorescence results of DCFH, CMFDA, FTC, Mitotracker Green, Annexin, DIBAC, and MitoPY 1 were collected using a 527/32 507LP filter. Fluorescence results of MitoSOX and HE were collected using a 700/54 665LP filter, and TMRM and PI fluorescence results were collected using a 586/42 560LP filter. The fluorescence of DAPI was collected using a 448/45 filter. The fluorescence of B665 was detected using 586/42 556LP and 780/60 735LP filters. The DAF-FM DA, DHR1,2,3, and FLUO-4 fluorescence were gathered using a 530/30 505LP filter.

BD FACSSuite software was used for data acquisition with the FACSVerse cytometer, and the FACSDiva 4.0 software was used for FACSAriaIII and LSR Fortessa X-20 cytometer data acquisition. Offline data analysis was performed using the FLOWJO V.10.1 software (FlowJo™ Software Version 10.1. Becton, Dickinson and Company, 2019; Ashland, OR, USA).

## 3. Results

Due to the lack of previous research regarding the OS profile of the human nasal epithelial cells, a complex experimental design was needed. Besides cell quantity adjustment, reactive concentration, and voltage, positive controls were used to ensure that each parameter was correctly determined, as explained below. Basal labelling, the positive control and the corresponding graphics for each parameter from the six healthy individuals included in the study are shown in the Supplementary Materials section.

### 3.1. Gating Strategy

**Figure 1:**
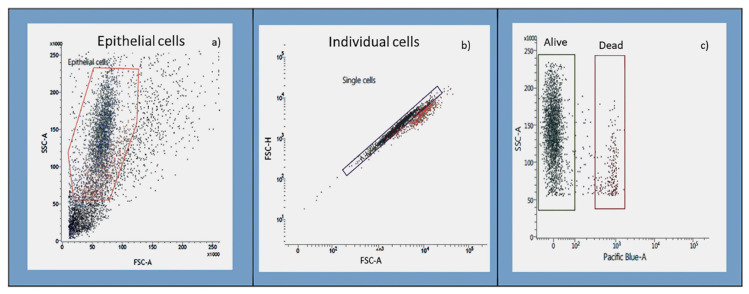
Gating strategy used to select the population of interest using a FACS Verse cytometer. (**a**) Nasal epithelial cells were selected by side scatter characteristic (SSC) and forward scatter characteristic (FSC) density plots. (**b**) A gate was applied to identify specific populations of individual cells. Each dot represents one nasal epithelial cell that passed through the cytometer laser. (**c**) Dead cells were identified and excluded from further analysis using either DAPI or PI.

Figure 1 shows the population gating strategy used for each parameter. Nasal epithelial cells were selected by morphology, as measured by forward scatter (FSC), a cell size indicator, and side scatter (SSC), which estimates the internal complexity of the cells (1A). After this, individual cells were selected from all nasal epithelial cells through height confrontation (high-FSC) in front of the FSC area (area-FSC), disregarding doublets (1B). Dead cells were excluded using DAPI or PI (1C).

### 3.2. ROS and RNS Generation

Supplementary Tables S1 and S2 show an example of intracellular peroxides and total O_2_^−^ levels detected after incubating the nasal epithelial cells with the respective fluorescent probes and inductors in positive controls. The gating strategy shown in Figure 1 was used, and dead cells were discarded using DAPI.

**Figure 2:**
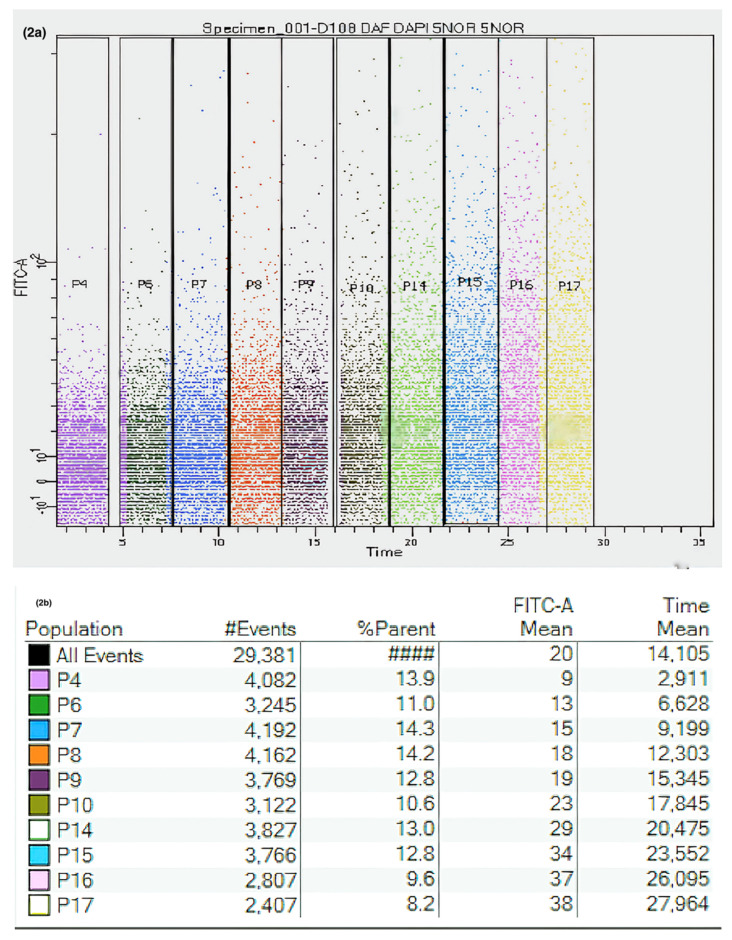
Kinetic measurements of nitric oxide (NO) assays using flow cytometry. (**a**) Dot blot acquired from kinetic measurements. Live nasal epithelial cells were gated according to FSC and SSC, and the gated events were plotted against a FITC-A channel (in this case, DAF-FM DA) and time. (**b**) Table showing gating percentages and fluorescence levels increasing over time.

**Figure 3:**
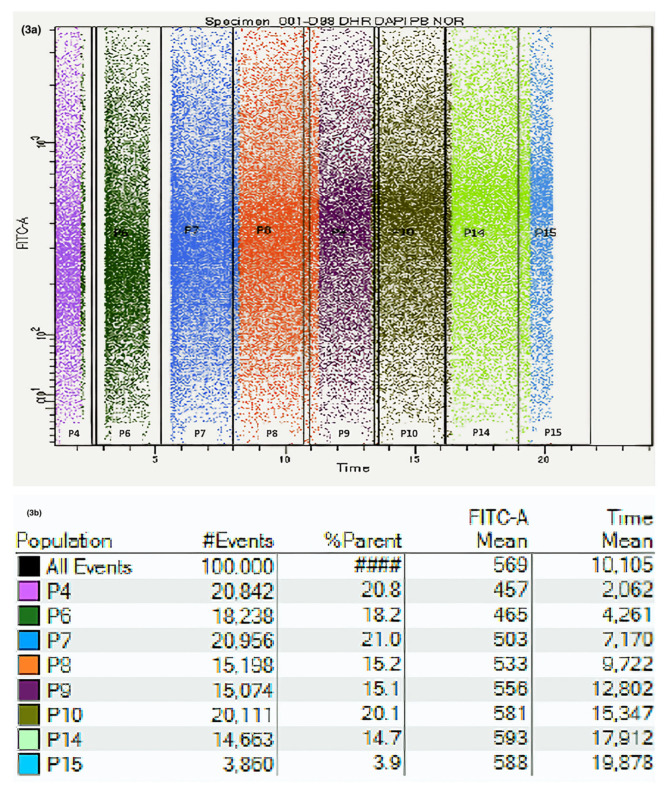
Flow cytometric analysis of peroxynitrite generation kinetics in nasal epithelial cells. (**a**) Dot blot acquired from kinetic measurements. Live nasal epithelial cells were gated according to FSC and SSC, and the gated events were plotted against a FITC-A channel (in this case DHR123) and time. Acquisition was paused at two points to add plumbagin (PB) (an O_2_^−^ provider) and NOR-1 (a NO inductor). (**b**) Table showing gating percentages and fluorescence levels increasing over time.

A kinetic strategy was designed for the real-time follow-up of intracellular NO (Supplementary Table S3) and ONOO^−^ generation (Supplementary Table S4). Single human nasal epithelial cells were selected based on morphology and DAPI exclusion. The strategies for the sequential gating of NO and ONOO^−^ generation are shown in Figure 2 and Figure 3, respectively.

### 3.3. GSH Detection

Supplementary Table S5 shows an example of total GSH levels after incubating the nasal epithelial cells with CMF and DEM as a positive control. The gating strategy shown in Figure 1 was used, and DAPI was used to identify dead cells.

### 3.4. Intracellular Ca^2+^ and Plasma Membrane Potential Detection

**Figure 4:**
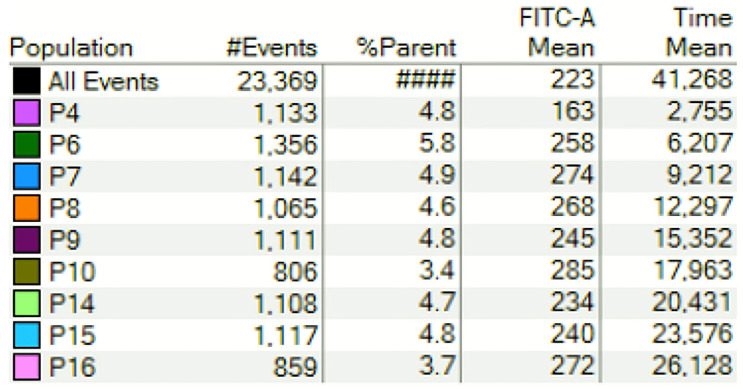
Flow cytometric analysis of intracellular calcium (iCa2+) generation kinetics in nasal epithelial cells. Intracellular calcium was measured using FLUO-4. Human nasal cells were gated accordingly to SSC and FSC. Dead cells were excluded using DAPI. Gating percentages and iCa2+ levels are shown.

A kinetic strategy was designed for the real-time follow-up of intracellular Ca^2+^ generation. Human nasal epithelial cells were selected based on morphology. After that, single cells were selected, and dead cells were discarded using DAPI. Gating percentages and fluorescence values for FLUO-4 are shown in Figure 4 and Supplementary Table S6.

Plasmatic membrane potential levels detected after incubating the nasal epithelial cells with DIBAC and t-BHP (positive controls) are shown in Supplementary Table S7. The gating strategy shown in Figure 1 was used, with PI used to identify any dead cells.

### 3.5. Mitochondrial Function

Supplementary Tables S8–S11 show the levels of mitochondrial membrane potential, mitochondrial mass, and mitochondrial H_2_O_2_ and O_2_^–^ detected after incubating the nasal epithelial cells with their respective fluorochromes and inductors for positive controls. The gating strategy in Figure 1 was used, and DAPI was used to identify dead cells.

### 3.6. Oxidative Damage in Lipids and Proteins Analysis

Supplementary Table S12 shows the carbonylated protein levels after incubating the nasal epithelial cells with FTC and menadione in the positive controls. The gating strategy in Figure 1 was used, and DAPI was used to identify dead cells.

**Figure 5:**
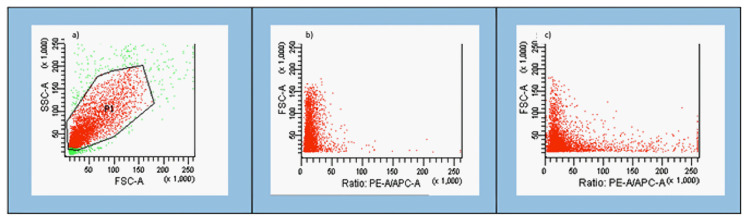
Evaluation of lipid peroxidation using BODIPY 665/676 C11. (**a**) Nasal epithelial cells were selected based on morphology and PI staining to exclude dead cells. (**b**) and (**c**) show the ratios of oxidised vs. reduced lipids.

Figure 5 shows the detection and gating strategy and the corresponding fluorescence values of the lipidic peroxidation levels in the human nasal epithelial cells after incubation with BODIPY665. Nasal epithelial cells were selected based on morphology after eliminating doublets, and fluorescence levels were determined for BODIPY665. Supplementary Table S13 shows the basal sample results and the control, which were previously incubated with t-BHP and then with BODIPY665.

### 3.7. Apoptosis and Cell Death Detection

**Figure 6:**
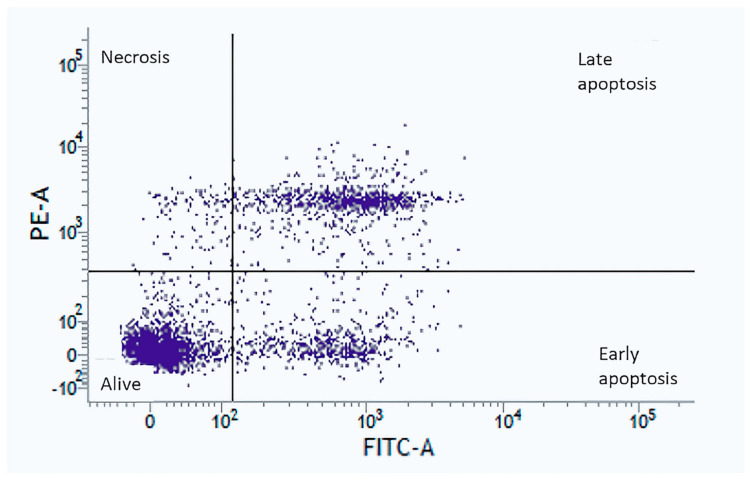
Apoptosis and cell death determination using Annexin V and propidium iodide (PI) staining. Nasal epithelial cells were selected based on morphology. Apoptosis status was determined by Annexin V staining. Cell death was determined by PI staining. Gating methods for Annexin V−/PI− (live), Annexin V+/PI− (early apoptosis), Annexin V−/PI+ (necrosis), and Annexin V+/PI+ (late apoptosis) are shown.

Figure 6 shows the levels of apoptosis and cell death of human nasal epithelial cells after Annexin V and PI staining. Nasal epithelial cells were selected based on morphology after eliminating doublets. Gating for Annexin V−/PI− (live), Annexin V+/PI− (early apoptosis), Annexin V−/PI+ (necrosis), and Annexin V+/PI+ (late apoptosis) populations are demonstrated.

## 4. Discussion

Numerous studies have shown the importance of OS in the physiopathology of diseases. Therefore, detailed knowledge of the association between OS and pathogenesis could be used to deepen the understanding of the disease’s pathophysiology and the discovery of new therapeutic targets that will allow the development of new drugs with clinical utility. Three approaches are used to measure OS: (i) the direct determination of ROS/RNS; (ii) the determination of oxidative damage to biomolecules (lipids, proteins, and nucleic acids); and (iii) the determination of enzymatic and non-enzymatic antioxidant systems [54]. Whichever method is used, OS is difficult to measure for several reasons. Firstly, sampling itself is a source of OS and must be carried out carefully to avoid OS artificial generation that could oxidise the biomolecules, giving rise to false results. The addition of a control group whose samples are manipulated in the same way as those of the patients would partially solve this problem, since the differences between groups could be attributed to changes related to the disease’s physiopathology. Secondly, many of the techniques used have a low sensitivity, which implies that a large number of cells must be used to obtain reliable results. This is a significant problem, mainly when often limited clinical samples are used. Thirdly, ROS/RNS have a very short half-life and it is therefore difficult to determine them accurately and precisely. To solve this problem, the indirect measurement of ROS/RNS was used by assessing the oxidative damage that these radicals cause in biomolecules [55,56].

Although many analytical techniques have been developed to measure OS over the years, there is still no gold standard. Methods such as electron spin resonance or nuclear magnetic resonance lack sensitivity and are unfeasible due to the short half-life of some ROS/RNS. Other methods, such as gas chromatography/mass spectrometry or high-performance liquid chromatography, require highly trained personnel. Recently, techniques based on spectrophotometry and enzyme assays have been developed. These methods have several advantages, such as being easy to perform, quantitative, and high-throughput and having a high sensitivity and the ability to be used with a wide variety of biological samples (serum, plasma, saliva, etc.). However, when analysing heterogeneous populations (e.g., blood), all these methods can lead to biased results as they are not able to separate the different cell populations, and therefore only overall results are obtained [55,56,57,58].

Flow cytometry has several advantages over previous methods in determining OS. It can measure OS in a large number of individual cells quickly and identify subpopulations within heterogeneous samples, providing qualitative results from a high number of individual cells from a particular subpopulation, instead of measuring the average of the total population. It also allows the analysis of multiple parameters from the same biological sample in a single experiment. Besides, sorting of subpopulations is possible, allowing further analysis by other methods of these sorted subpopulations. Our group aims to characterise the role of OS in rare respiratory diseases, such as PCD, to study its potential implication in disease pathophysiology. As sample yield obtained from biopsies is often a limiting factor when studying the affected tissue in respiratory diseases, we developed a novel method based on flow cytometry to determine fourteen parameters related to cell metabolism using a small amount of sample. To our knowledge, this is the first use of flow cytometry to assess OS levels in human nasal epithelial cells and could represent a new procedure for the study of respiratory diseases in which nasal epithelial cells are the affected tissue.

It has been described that mitochondria, which are the main cellular organelles involved in the production of ROS/RNS, are affected in some respiratory diseases [59]. Therefore, a new protocol to study the mitochondrial mass, mitochondrial membrane potential, and mitochondrial production of H_2_O_2_ and O_2_^−^ in human nasal epithelial cell samples was set up. Moreover, a method to measure the general oxidative status of these cells and their ROS and RNS production, including intracellular peroxides, O_2_^−^, and ONOO^−^, was developed. As some respiratory diseases are related to inflammation, which is also related to OS, this methodology allowing us to study reactive species production in affected tissues could be advantageous [1,11]. In addition, the study of intracellular Ca^2+^ and plasma membrane potential provides investigators with necessary information regarding cell metabolism, which could provide insight into various diseases. In our study, Fluo-4 has been used to measure intracellular Ca^2+^ levels. Single-wavelength dyes, such as Fluo-4, cannot provide quantitative data as the variability of dye concentration and/or photobleaching influence the emission intensities. Consequently, using Fluo-4 only the presence/absence of intracellular Ca^2+^ can be determined, and the use of dual-wavelength dyes, such as Fura-2 AM, that allows for accurate quantification of intracellular Ca^2+^ concentration, is only limited by the C^2+^ response of Fura-2 [60]. Finally, mitochondrial GSH, one of the most important non-enzymatic defences against OS, can be studied relatively quickly and cheaply using this protocol [9].

Additionally, this work allows the study of OS damage in biomolecules, such as proteins and lipids [1] [10]. Combined with the study of apoptosis and cellular death, this provides a complete profile of the effect of oxidative stress on human nasal epithelial cells, which make up part of the affected tissues in some respiratory diseases, such as PCD [61]. Finally, a great advantage of this method is that a comprehensive set of OS parameters regarding different respiratory diseases could be studied in future research using tiny samples and non-invasive sampling techniques.

## 5. Limitations

This article was written to show a laboratory protocol to measure OS parameters in nasal epithelial cells. The study was performed using samples from a small number of healthy individuals, so it is not intended to draw conclusions on OS status, which should be done in future research. As an example, the fluorescence values obtained in the samples (Supplementary Tables S1–S13) are shown.

## 6. Conclusions

We have established a method based on flow cytometry to study a comprehensive set of OS parameters in nasal epithelial cells that could be useful in research on respiratory diseases. This method has the additional advantage of using small amounts of sample and a non-invasive sampling technique.

## Figures and Tables

**Table 1:** Summary of the flow cytometry methods.

Reactive Species	Measurement	Stock Concentration	Storage Temperature	Final Concentration	Flow Cytometer	Excitation Laser	Detector	Cell Number	Total Volume	Incubation Time	Incubation Temperature
**DIBAC**	Plasma membrane potential	100 µM	−20 °C	1.2 µM	FACS Verse	Blue (488 nm)	527/32507 LP	8.000	250 µL	20 min	37 °C
**FLUO-4**	Intracellular Ca^2+^	50 µM	−20 °C	0.5 µM	LSR Fortessa X-20	Blue (488 nm)	530/30505LP	8.000	250 µL	20 min	37 °C
**CMFDA**	Reduced thiols (GSH)	10 µM	−20 °C	25 nM	FACS Verse	Blue (488 nm)	527/32507 LP	8.000	250 µL	20 min	37 °C
**DAF-FMDA**	NO	1.25 mM	−20 °C	1 µM	LSR Fortessa X-20	Blue (488 nm)	530/30505LP	8.000	250 µL	20 min	37 °C
**DCF**	Intracellular peroxides	1 mg/mL	−20 °C	2.5 µg/mL	FACS Verse	Blue (488 nm)	527/32507 LP	8.000	250 µL	20 min	37 °C
**MitoSOX**	Motochondrial O_2_^−^	0.5 mM	−20 °C	640 nM	FACS Verse	Blue (488 nm)	700/54665LP	8.000	250 µL	20 min	37 °C
**Mitotracker Green**	Mitochondrial mass	10 µM	−20 °C	78 nM	FACS Verse	Blue (488 nm)	527/32507 LP	8.000	250 µL	20 min	37 °C
**BODIPY 665/676**	Oxidized/reduced lipid ratio	1 mM	−20 °C	800 nM	FACS Aria III	Blue (488 nm) Red (635 nm)	586/42556LP and 780/60735LP	12.000	250 µL	30 min	37 °C
**TMRM**	Mitochondrial Ψm	240 µM	−20 °C	600 nM	FACS Verse	Blue (488 nm)	586/42560LP	8.000	250 µL	20 min	37 °C
**DHR 123**	ONOO^−^	5 mM	−20 °C	100 µM	LSR Fortessa X-20	Blue (488 nm)	530/30505LP	12.000	250 µL	20 min	37 °C
**HE**	O_2_^−^	1 mg/mL	−20 °C	2.5 µg/mL	FACS Verse	Blue (488 nm)	700/54665LP	8.000	250 µL	20 min	37 °C
**FTC**	Protein carbonylation	1 mM	−20 °C	800 nM	FACS Verse	Blue (488 nm)	527/32507 LP	8.000	250 µL	20 min	37 °C
**MitoPY**	Mitochondrial H_2_O_2_	1 mM	−20 °C	4 µM	FACS Verse	Blue (488 nm)	527/32507 LP	8.000	250 µL	20 min	37 °C
**DAPI**	Cell death	1 mg/mL	−20 °C	800 ng/mL	FACS Verse	Violet (405 nm)	448/45	8.000	250 µL	20 min	37 °C
**PI**	Cell death	1 mg/mL	4 °C	8 µg/mL	FACS Verse	Blue (488 nm)	586/42560LP	12.000	100 µL	15 min	RT
**Annexin V**	Apoptosis	-	4 °C	-	FACS Verse	Blue (488 nm)	527/32507 LP	12.000	100 µL	15 min	RT

**Table 2:** Summary of cytometry methods for positive controls.

Reactive Species	Measurement	Inductor	Stock Concentration	Final Concentration	Storage Temperature	Inductor Incubation Time	Inductor Incubation Temperature	Reactive Incubation Time	Reactive Incubation Temperature
**DIBAC**	Plasmatic membrane potential	t-BHP	7.7 mM	100 μM	4 °C	15 min	37 °C	30 min	37 °C
**FLUO-4**	Intracellular Ca^2+^	Ionomycin	1.338 mM	50 μM	−20 °C	Kynetics		Kynetics	
**CMFDA**	Reduced thiols	DEM	-	20 mM	−20 °C	90 min	37 °C	30 min	37 °C
**DAF-FM DA**	NO	NOR-1	1 mg/mL	16 μg/mL	−20 °C	Kynetics		Kynetics	
**DCF**	Intracellular peroxides	t-BHP	7.7 mM	100 μM	4 °C	15 min	37 °C	30 min	37 °C
**MitoSOX**	Mitochondrial O_2_^−^	PB	2.8 mg/mL	2.24 μg/mL	−20 °C	15 min	37 °C	30 min	37 °C
**BODIPY 665/676**	Oxidized/reduced lipids ratio	t-BHP	7.7 mM	100 μM	4 °C	15 min	37 °C	30 min	37 °C
**TMRM**	Mitochondrial Ψ_m_	FCCP	10 mM	52 μM	−20 °C	15 min	37 °C	30 min	37 °C
**DHR 123**	ONOO-	NOR-1 and PB	1 mg/mL and 2.8 mg/mL	16 μg/mL and 2.24 μg/mL	−20 °C	Kynetics		Kynetics	
**HE**	O_2_^−^	PB	2.8 mg/mL	2.24 μg/mL	−20 °C	15 min	37 °C	30 min	37 °C
**FTC**	Protein carbonylation	Menadione	10 mg/mL	1 mM	−20 °C	60 min	37 °C	30 min	37 °C
**MitoPY**	Mitochondrial H_2_O_2_	t-BHP	7.7 mM	100 μM	4 °C	15 min	37 °C	30 min	37 °C

## Data Availability

The data presented in this study are available on request from the corresponding author. The data are not publicly available due to privacy/ethical restrictions.

## Supplementary Materials

The following are available online at https://www.mdpi.com/2077-0383/10/6/1172/s1, Table S1: Basal labeling and the corresponding positive control for intracellular H_2_O_2_ from six healthy individuals; Table S2: Basal labeling and the corresponding positive control for O_2_^−^ from six healthy individuals; Table S3: Basal labeling and the corresponding positive control for nitric oxide (NO) from six healthy individuals; Table S4: Basal labeling and the corresponding positive control for peroxynitrite (ONOO^−^) from six healthy individuals; Table S5: Basal labeling and the corresponding positive control for GSH from six healthy individuals; Table S6: Basal labeling and the corresponding positive control for intracellular Calcium (iCa_2_^+^) from six healthy individuals; Table S7: Basal labeling and the corresponding positive control for plasmatic membrane potential (PMP) from six healthy individuals; Table S8: Basal labeling and the corresponding positive control for mitochondrial membrane potential (∆ψm) from six healthy individuals; Table S9: Basal labeling and the corresponding positive control for mitochondrial H_2_O_2_ (mt H_2_O_2_) from six healthy individual; Table S10: Basal labeling and the corresponding positive control for mitochondrial O_2_^−^ (mtO_2_^−^) from six healthy individuals; Table S11: Labeling for mitochondrial mass from six healthy individuals; Table S12: Basal labeling and the corresponding positive control for carbonylated proteins from six healthy individuals; Table S13: Basal labeling and the corresponding positive control for the oxidized/reduced lipid ratio (ox/red lipid) from six healthy individuals.
