# Supplementary material for: New Laboratory Protocol to Determine the Oxidative Stress Profile of Human Nasal Epithelial Cells Using Flow Cytometry
Source: J Clin Med. 2021 Mar 11;10(6):1172. doi: 10.3390/jcm10061172 (PMC7998408; doi:10.3390/jcm10061172)

Supplementary materials

Reagents. The fluorescent probes Bis-(Dibutylbarbituric Acid) Trimethine Oxonol (DIBAC), FLUO-4, 5-chloromethyl fluorescein diacetate (CMFDA), 4-Amino-5-Methylamino-2'7'-Difluorescein Diacetate (DAF-FM DA), 2'7'-dichlorodihydrofluorescein (DCFH), MitoSOX^{TM} Red (MitoSOX), Mitotracker Green, BODIPY 665/676 C 11 (B665), Tetramethylrhodamine (TMRM) and dihydrorhodamine 1,2,3 (DHR1,2,3), were from Molecular Probes (Eugene, OR). Dihydroethidium (HE), Propidium Iodide (PI), fluorescein 5-thiosemicarbazide (FTC), 4', 6'-Diamidino-2-phenylindole dihydrochloride (DAPI), and Mitochondria peroxy yellow 1 (MitoPY 1) were from Sigma (San Luis, MO). Anexine V was purchased from Inmunostep (Salamanca, Spain). The inductors tert-Butyl hydroperoxide (t-BHP), Ionomycin ionophore, Diethyl maleate (DEM), Plumbagin (PB), Carbonyl cyanide 4-(trifluoromethoxy)phenylhydrazone (FCCP), and Menadione were from Sigma (San Luis, MO). NOR-1 was from Santa Cruz Biotechnology (Dallas, TX).

Step-by-step protocol

LABORATORY PROTOCOL TO MEASURE THE OXIDATIVE STRESS PROFILE OF HUMAN NASAL EPITHELIAL CELLS BY FLOW CYTOMETRY

To obtain a nasal epithelial biopsy, insert a cytology brush into the patient's nostril and gently brushed the nasal epithelium of the middle meatus for about 20 seconds.

Put the cytology brush containing the nasal epithelial biopsy in a 15 ml sterile tissue culture tube containing Medium199 supplemented with Hanks' salts, L-glutamine, 25 mM HEPES 1% penicillin/streptomycin. Take it to the lab.

Gently shake the brush to lose cells into the medium. Filter using 50 µm CellTrics® filters (25004-0042-2317 Sysmex) to isolate individual cells from cell debris and aggregates.

Count cells using a FACS Verse Cytometer. At this step, you know the number of cells per µl in your sample.

Unless otherwise stated perform all incubations in the dark.

PLASMA MEMBRANE POTENTIAL

BASAL

1. Take enough sample volume to have 8000 cells.
2. Add 3 µl of DIBAC (stock 100µM) to a final concentration of 1.2µM.
3. Add 2µl of PI (stock 1mg/ml) to a final concentration of 8µg/ml.
4. Add Medium199 to a total volume of 250µl.
5. Incubate 20min at 37ºC.
6. Measure fluorescence using a FACS Verse Cytometer (Excitation laser 488 nm; Detector: 527/32507 LP).

POSITIVE CONTROL

1. Take enough sample volume to have 8000 cells.
2. Add 3.25µl of t-BHP (stock 7.7mM) to a final concentration of 100µM.
3. Incubate 15min at 37ºC.
4. Add 3 µl of DIBAC (stock 100µM) to a final concentration of 1.2µM.
5. Add 2µl of PI (stock 1mg/ml) to a final concentration of 8µg/ml.
6. Incubate 30min at 37ºC.
7. Measure fluorescence using a FACS Verse Cytometer (Excitation laser 488 nm; Detector: 527/32507 LP).

INTRACELLULAR Ca^{2+}

BASAL

1. Take enough sample volume to have 8000 cells.
2. Add 2.5µl of FLUO-4 (stock 50µM) to a final concentration of 0.5µM.
3. Add 0.2µl of DAPI (stock 1mg/ml) to a final concentration of 800ng/ml.
4. Add Medium199 to a total volume of 250µl.
5. Incubate 20min at 37ºC.

6. Measure fluorescence using a LSR Fortessa X-20 Cytometer (Excitation laser 488 nm; Detector: 530/30505 LP).

POSITIVE CONTROL-KINETICS

1. Take enough sample volume to have 12000 cells.
2. Add 2.5µl of FLUO-4 (stock 50µM) to a final concentration of 0.5µM.
3. Add 0.2µl of DAPI (stock 1mg/ml) to a final concentration of 800ng/ml.
4. Add Medium199 to a total volume of 250µl.
5. Incubate 20min at 37ºC.
6. Start fluorescence measurement using a LSR Fortessa X-20 Cytometer (Excitation laser 488 nm; Detector: 530/30505 LP).
7. Pause acquisition process
8. Add 9.34µl of Ionomycin (stock 1.338mM) to a final concentration of 50µM
9. Continue the acquisition process until 600s.

REDUCED THIOLS (GSH)

BASAL

1. Take enough sample volume to have 8000 cells.
2. Add 0.625µl of CMF-DA (stock 10µM) to a final concentration of 25nM.
3. Add 0.2µl of DAPI (stock 1mg/ml) to a final concentration of 800ng/ml.
4. Add Medium199 to a total volume of 250µl.
5. Incubate 20min at 37ºC.
6. Measure fluorescence using a FACS Verse Cytometer (Excitation laser 488 nm; Detector: 527/32507 LP).

POSITIVE CONTROL

1. Take enough sample volume to have 8000 cells.
2. Add 20 mM DEM
3. Incubate 90min at 37ºC
4. Add 0.625µl of CMF-DA (stock 10µM) to a final concentration of 25nM.
5. Add 0.2µl of DAPI (stock 1mg/ml) to a final concentration of 800ng/ml.
6. Incubate 30min at 37ºC.
7. Measure fluorescence using a FACS Verse Cytometer (Excitation laser 488 nm; Detector: 527/32507 LP).

Nitric Oxide

BASAL

1. Take enough sample volume to have 8000 cells.
2. Add 0.2µl of DAF-FMDA (stock 1.25mM) to a final concentration of 1µM.
3. Add 0.2µl of DAPI (stock 1mg/ml) to a final concentration of 800ng/ml.
4. Add Medium199 to a total volume of 250µl.

5. Incubate 20min at 37ºC.

6. Measure fluorescence using a LSR Fortessa X-20 Cytometer (Excitation laser 488 nm; Detector: 530/30505 LP).

POSITIVE CONTROL-KINETICS

1. Take enough sample volume to have 12000 cells.

2. Add 0.2µl of DAF-FMDA (stock 1.25mM) to a final concentration of 1µM.

3. Add 0.2µl of DAPI (stock 1mg/ml) to a final concentration of 800ng/ml.

4. Add Medium199 to a total volume of 250µl.

5. Incubate 20min at 37ºC.

6. Start fluorescence measurement using a LSR Fortessa X-20 Cytometer (Excitation laser 488 nm; Detector: 530/30505 LP).

7. Pause acquisition process

8. Add 4µl of NOR-1 (stock 1mg/ml) to a final concentration of 16µg/ml

9. Continue the acquisition process until 300s.

INTRACELLULAR PEROXIDES

BASAL

1. Take enough sample volume to have 8000 cells.

2. Add 0.625µl of DCF (stock 1mg/ml) to a final concentration of 2.5µg/ml.

3. Add 0.2µl of DAPI (stock 1mg/ml) to a final concentration of 800ng/ml.

4. Add Medium199 to a total volume of 250µl.

5. Incubate 20min at 37ºC.

6. Measure fluorescence using a FACS Verse Cytometer (Excitation laser 488 nm; Detector: 527/32507 LP).

POSITIVE CONTROL

1. Take enough sample volume to have 8000 cells.

2. Add 3.25µl of t-BHP (stock 7.7mM) to a final concentration of 100µM

3. Incubate 15min at 37ºC

4. Add 0.625µl of DCF (stock 1mg/ml) to a final concentration of 2.5µg/ml.

5. Add 0.2µl of DAPI (stock 1mg/ml) to a final concentration of 800ng/ml.

6. Incubate for 30min at 37ºC.

7. Measure fluorescence using a FACS Verse Cytometer (Excitation laser 488 nm; Detector: 527/32507 LP).

MITOCHONDRIAL O_2^-

BASAL

1. Take enough sample volume to have 8000 cells.

2. Add 0.32µL of MitoSOX (stock 0.5mM) to a final concentration of 640nM.

3. Add 0.2µl of DAPI (stock 1mg/ml) to a final concentration of 800ng/ml.

4. Add Medium199 to a total volume of 250µl.
5. Incubate 20min at 37ºC.
6. Measure fluorescence using a FACS Verse Cytometer (Excitation laser 488 nm; Detector: 700/54665 LP).

POSITIVE CONTROL

1. Take enough sample volume to have 8000 cells.
2. Add 0.2µl of PB (stock 2.8mg/ml) to a final concentration of 2.24µg/ml
3. Incubate 15min at 37ºC
4. Add 0.32µL of MitoSOX (stock 0.5mM) to a final concentration of 640nM.
5. Add 0.2µl of DAPI (stock 1mg/ml) to a final concentration of 800ng/ml.
6. Incubate 30min at 37ºC.
7. Measure fluorescence using a FACS Verse Cytometer (Excitation laser 488 nm; Detector: 700/54665 LP).

MITOCHONDRIAL MASS

1. Take enough sample volume to have 8000 cells.
2. Add 1.95µL of Mitotracker Green (stock 10µM) to a final concentration of 78nM.
3. Add 0.2µl of DAPI (stock 1mg/ml) to a final concentration of 800ng/ml.
4. Add Medium199 to a total volume of 250µl.
5. Incubate 20min at 37ºC.
6. Measure fluorescence using a FACS Verse Cytometer (Excitation laser 488 nm; Detector: 527/32507 LP).

OXIDIZED/REDUCED LIPID RATIO

BASAL

1. Take enough sample volume to have 12000 cells.
2. Add 0.2µl of BODIPY665/676 (stock 1mM) to a final concentration of 800nM.
3. Add 0.2µl of DAPI (stock 1mg/ml) to a final concentration of 800ng/ml.
4. Add Medium199 to a total volume of 250µl.
5. Incubate 30min at 37ºC.
6. Measure fluorescence using a FACS Aria III Cytometer (Excitation laser 488 nm and 635 nm; Detector: 588/42556 LP and 780/60735 LP).

POSITIVE CONTROL

1. Take enough sample volume to have 12000 cells.
2. Add 3.25µl of t-BHP (stock 7.7mM) to a final concentration of 100µM
3. Incubate 15min at 37ºC
4. Add 0.2µl of BODIPY665/676 (stock 1mM) to a final concentration of 800nM.
5. Add 0.2µl of DAPI (stock 1mg/ml) to a final concentration of 800ng/ml.
6. Incubate 30min at 37ºC.

7. Measure fluorescence using a FACS Aria III Cytometer (Excitation laser 488 nm and 635 nm; Detector: 588/42556 LP and 780/60735 LP).

MITOCHONDRIAL MEMBRANE POTENTIAL

BASAL

1. Take enough sample volume to have 8000 cells.
2. Add 0.625µl of TMRM (stock 240µM) to a final concentration of 600nM.
3. Add 0.2µl of DAPI (stock 1mg/ml) to a final concentration of 800ng/ml.
4. Add Medium199 to a total volume of 250µl.
5. Incubate 20min at 37ºC.
6. Measure fluorescence using a FACS Verse Cytometer (Excitation laser 488 nm; Detector: 586/42560 LP).
7.

POSITIVE CONTROL

1. Take enough sample volume to have 8000 cells.
2. Add 1.3µl of FCCP (stock 10mM) to a final concentration of 52µM
3. Incubate 15min at 37ºC
4. Add 0.625µl of TMRM (stock 240µM) to a final concentration of 600nM.
5. Add 0.2µl of DAPI (stock 1mg/ml) to a final concentration of 800ng/ml.
6. Incubate 30min at 37ºC.
7. Measure fluorescence using a FACS Verse Cytometer (Excitation laser 488 nm; Detector: 586/42560 LP).

Peroxinitrites ($ONOO^-$)

BASAL

1. Take enough sample volume to have 12000 cells.
2. Add 5µl of DHR1,2,3 (stock 5mM) to a final concentration of 100µM.
3. Add 0.2µl of DAPI (stock 1mg/ml) to a final concentration of 800ng/ml.
4. Add Medium199 to a total volume of 250µl.
5. Incubate 20min at 37ºC.
6. Measure fluorescence using a LSR Fortessa X-20 Cytometer (Excitation laser 488 nm; Detector: 530/30505 LP).

POSITIVE CONTROL-KINETICS

1. Take enough sample volume to have 12000 cells.
2. Add 5µl of DHR1,2,3 (stock 5mM) to a final concentration of 100µM.
3. Add 0.2µl of DAPI (stock 1mg/ml) to a final concentration of 800ng/ml.
4. Add Medium199 to a total volume of 250µl.
5. Incubate 20min at 37ºC.
7. Start fluorescence measurement using a LSR Fortessa X-20 Cytometer (Excitation laser 488 nm; Detector: 530/30505 LP).

6. Pause acquisition process
7. Add 4µl of NOR-1 (stock 1mg/ml) to a final concentration of 16µg/ml
8. Continue the acquisition process
9. Pause acquisition process
10. Add 0.2µl of PB (stock 2.8mg/ml) to a final concentration of 2.24µg/ml
11. Continue the acquisition process until 200s.

Superoxide anion (O_2^-)

BASAL

1. Take enough sample volume to have 8000 cells.
2. Add 0.625µL of HE (stock 1mg/ml) to a final concentration of 2.5µg/ml.
3. Add 0.2µl of DAPI (stock 1mg/ml) to a final concentration of 800ng/ml.
4. Add Medium199 to a total volume of 250µl.
5. Incubate 20min at 37ºC.
6. Measure fluorescence using a FACS Verse Cytometer (Excitation laser 488 nm; Detector: 700/54665 LP).

POSITIVE CONTROL

1. Take enough sample volume to have 8000 cells.
2. Add 0.2µl of PB (stock 2.8mg/ml) to a final concentration of 2.24µg/ml
3. Incubate 15min at 37ºC
4. Add 0.625µL of HE (stock 1mg/ml) to a final concentration of 2.5µg/ml.
5. Add 0.2µl of DAPI (stock 1mg/ml) to a final concentration of 800ng/ml.
6. Incubate 30min at 37ºC.
7. Measure fluorescence using a FACS Verse Cytometer (Excitation laser 488 nm; Detector: 700/54665 LP).

PROTEIN CARBONYLATION

BASAL

1. Take enough sample volume to have 8000 cells.
2. Add 0.2µL of FTC (stock 1mM) to a final concentration of 800nM.
3. Add 0.2µl of DAPI (stock 1mg/ml) to a final concentration of 800ng/ml.
4. Add Medium199 to a total volume of 250µl.
5. Incubate 20min at 37ºC.
6. Measure fluorescence using a FACS Verse Cytometer (Excitation laser 488 nm; Detector: 527/32507 LP).

POSITIVE CONTROL

1. Take enough sample volume to have 8000 cells.
2. Add Menadione (stock 10mg/ml) to a final concentration of 1mM
3. Incubate 60min at 37ºC

4. Add 0.2µL of FTC (stock 1mM) to a final concentration of 800nM.
5. Add 0.2µl of DAPI (stock 1mg/ml) to a final concentration of 800ng/ml.
6. Incubate 30min at 37ºC.
7. Measure fluorescence using a FACS Verse Cytometer (Excitation laser 488 nm; Detector: 527/32507 LP).

MITOCHONDRIAL H_2O_2

BASAL

1. Take enough sample volume to have 8000 cells.
2. Add 1µL of MitoPY (stock 1mM) to a final concentration of 4µM.
3. Add 0.2µl of DAPI (stock 1mg/ml) to a final concentration of 800ng/ml.
4. Add Medium199 to a total volume of 250µl.
5. Incubate 20min at 37ºC.
6. Measure fluorescence using a FACS Verse Cytometer (Excitation laser 488 nm; Detector: 527/32507 LP).

POSITIVE CONTROL

1. Take enough sample volume to have 12000 cells.
2. Add 3.25µl of t-BHP (stock 7.7mM) to a final concentration of 100µM
3. Incubate 15min at 37ºC
4. Add 1µL of MitoPY (stock 1mM) to a final concentration of 4µM.
5. Add 0.2µl of DAPI (stock 1mg/ml) to a final concentration of 800ng/ml.
6. Incubate 30min at 37ºC.
7. Measure fluorescence using a FACS Verse Cytometer (Excitation laser 488 nm; Detector: 527/32507 LP).

APOPTOSIS

1. Take enough sample volume to have 12000 cells.
2. Add 5 µl of Annexin V and 5 µl of PI
3. Add Annexin V-binding buffer (previously diluted /10 in PBS) to a total volume of 100 µl.
4. Incubate 15min at RT.
5. After incubation, add 300 µl of 1/10 dilution of Annexin V-binging buffer before reading fluorescence.
6. Measure fluorescence using a FACS Verse Cytometer (Excitation laser 488 nm; Detector: 527/32507 LP).

Table S1. Basal labeling and the corresponding positive control for intracellular H_2O_2 from six healthy individuals. Arbitrary fluorescence units (FU) for each labeling are shown. Fluorescence means and standard deviation (SD) of basal and induced values are shown. Significant statistical differences are observed between basal and induced measurements (Paired t-test; $p<0.0001$).

**Sample**	**Basal H_2O_2 (FU)**	**t-BHP-induced H_2O_2 (FU)**
1	573	1010
2	524	943
3	386	816
4	449	853
5	543	1012
6	599	1165
Mean	512	966
SD	80	126

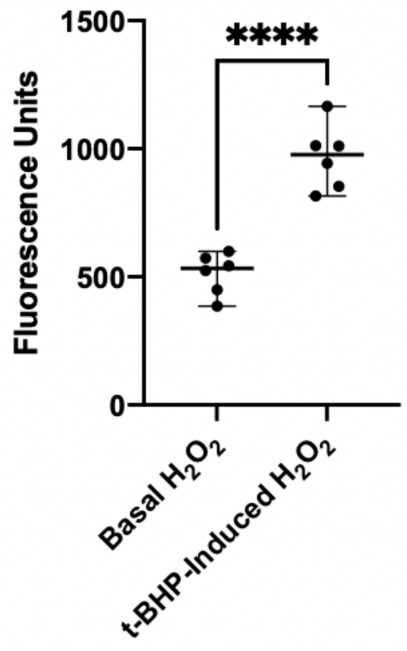

Table S2. Basal labeling and the corresponding positive control for O_2^- from six healthy individuals. Arbitrary fluorescence units (FU) for each labeling are shown. Fluorescence means and standard deviation (SD) of basal and induced values are shown. Significant statistical differences are observed between basal and induced measurements (Paired t-test; $p<0.001$).

Sample	Basal O_2^-(FU)	Plumbagin-induced O_2^- (FU)
1	694	1101
2	698	943
3	849	816
4	724	853
5	370	1012
6	607	1165
Mean	657	1130
SD	160	157

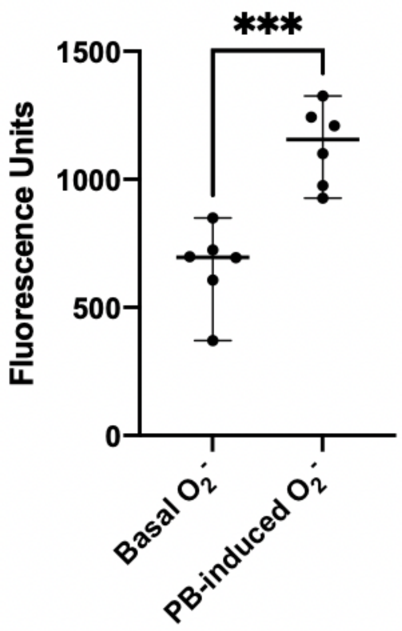

Table S3. Basal labeling and the corresponding positive control for nitric oxide (NO) from six healthy individuals. Arbitrary fluorescence units (FU) for each labeling are shown. Fluorescence means and standard deviation (SD) of basal and induced values are shown. Significant statistical differences are observed between basal and induced measurements (Paired t-test; $p<0.01$).

Sample	Basal NO (FU)	NOR1-induced NO (FU)
1	42	245
2	75	159
3	144	378
4	141	256
5	59	127
6	66	135
Mean	114	217
SD	70	96

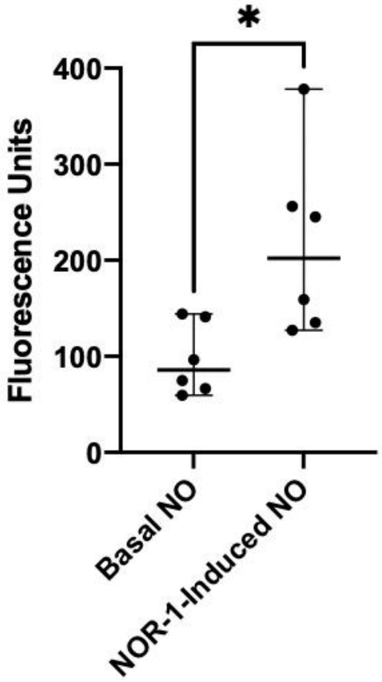

Table S4. Basal labeling and the corresponding positive control for peroxynitrite ($ONOO^-$) from six healthy individuals. Arbitrary fluorescence units (FU) for each labeling are shown. Fluorescence means and standard deviation (SD) of basal and induced values are shown. Significant statistical differences are observed between basal and induced measurements (Paired t-test; $p<0.001$).

Sample	Basal $ONOO^-$ (FU)	NOR1-induced $ONOO^-$ (FU)
1	41	146
2	39	184
3	45	1445
4	10	45
5	32	168
6	53	176
Mean	37	144
SD	15	51

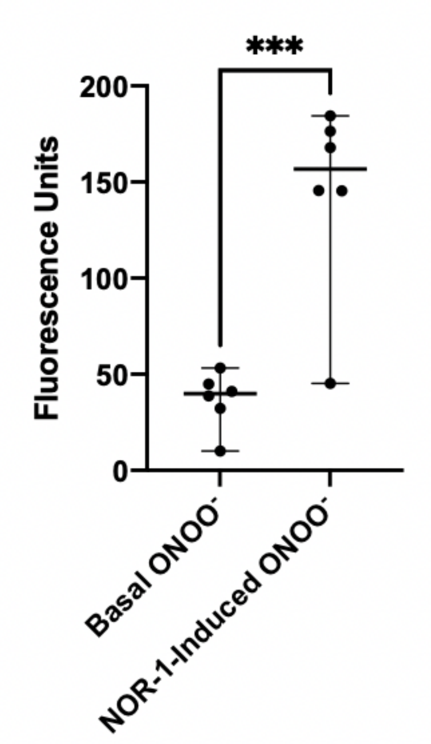

Table S5. Basal labeling and the corresponding positive control for GSH from six healthy individuals. Arbitrary fluorescence units (FU) for each labeling are shown. Fluorescence means and standard deviation (SD) of basal and induced values are shown. Significant statistical differences are observed between basal and induced measurements (Paired t-test; p<0.0001).

Sample	Basal GSH (FU)	DEM-depleted GSH
1	1216	261
2	1418	287
3	1818	314
4	1811	275
5	1369	325
6	1891	246
Mean	1587	285
SD	286	30

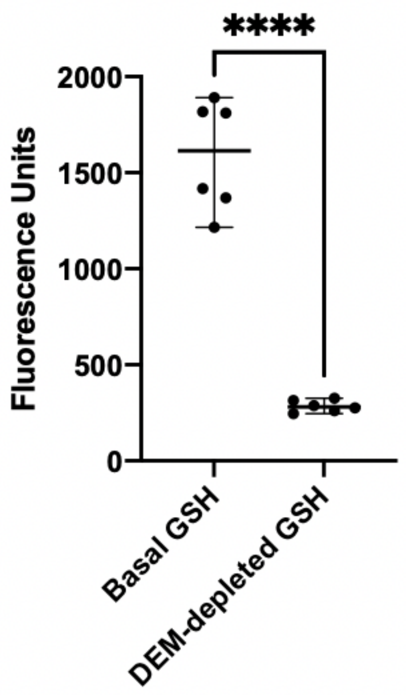

Table S6. Basal labeling and the corresponding positive control for intracellular Calcium (iCa$_2^+$) from six healthy individuals. Arbitrary fluorescence units (FU) for each labeling are shown. Fluorescence means and standard deviation (SD) of basal and induced values are shown. Significant statistical differences are observed between basal and induced measurements (Paired t-test; $p<0.01$).

Sample	Basal iCa$_2^+$ (FU)	NOR1-induced iCa$_2^+$ (FU)
1	42	94
2	23	64
3	32	73
4	23	57
5	47	92
6	93	162
Mean	43	90
SD	26	38

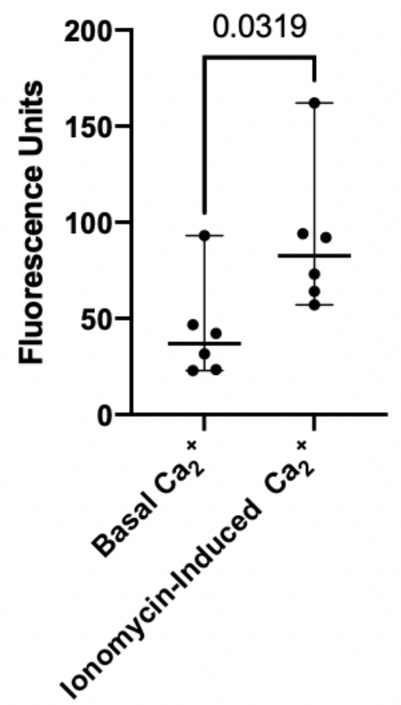

Table S7. Basal labeling and the corresponding positive control for plasmatic membrane potential (PMP) from six healthy individuals. Arbitrary fluorescence units (FU) for each labeling are shown. Fluorescence means and standard deviation (SD) of basal and induced values are shown. Significant statistical differences are observed between basal and induced measurements (Paired t-test; p<0.0001).

Sample	Basal PMP (FU)	t-BHP-induced PMP (FU)
1	740	115
2	749	143
3	1012	247
4	790	190
5	1058	249
6	988	220
Mean	889	194
SD	145	55

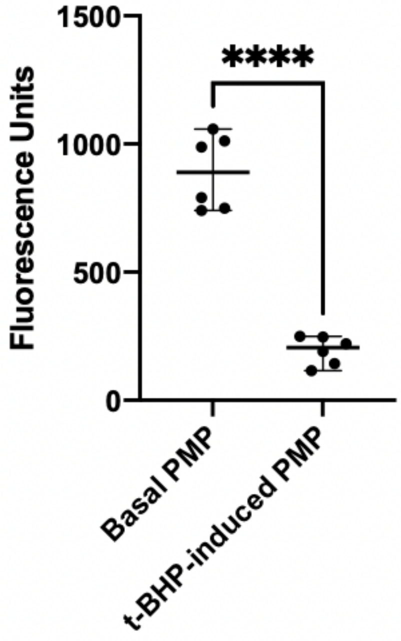

Table S8. Basal labeling and the corresponding positive control for mitochondrial membrane potential (Δψm) from six healthy individuals. Arbitrary fluorescence units (FU) for each labeling are shown. Fluorescence means and standard deviation (SD) of basal and induced values are shown. Significant statistical differences are observed between basal and induced measurements (Paired t-test; p<0.001).

Sample	Basal (Δψm) (FU)	TMRM-induced (Δψm) (FU)
1	1763	2540
2	948	1810
3	1249	2427
4	1290	2430
5	1519	2501
6	1417	2498
Mean	1364	2368
SD	275	277

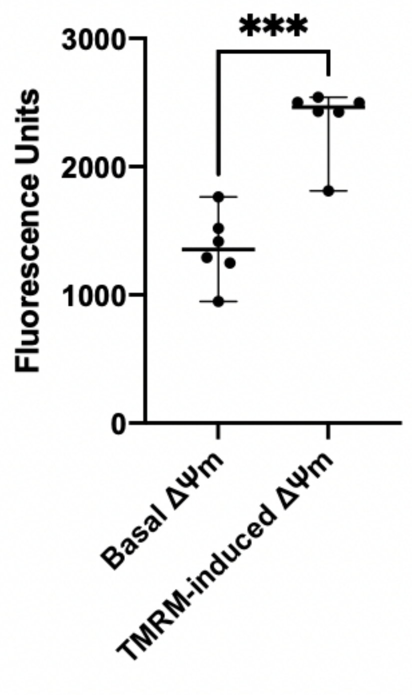

Table S9. Basal labeling and the corresponding positive control for mitochondrial H_2O_2 (mt H_2O_2) from six healthy individuals. Arbitrary fluorescence units (FU) for each labeling are shown. Fluorescence means and standard deviation (SD) of basal and induced values are shown. Significant statistical differences are observed between basal and induced measurements (Paired t-test; $p<0.001$).

**Sample**	**Basal mtH_2O_2 (FU)**	**t-BHP-induced mtH_2O_2 (FU)**
1	473	652
2	494	657
3	515	690
4	502	659
5	479	649
6	476	628
Mean	490	655
SD	17	20

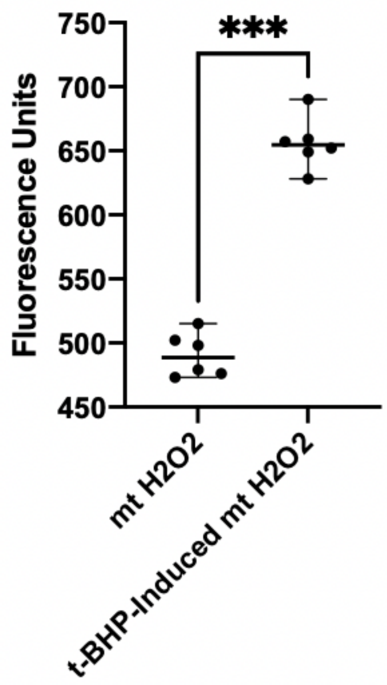

Table S10. Basal labeling and the corresponding positive control for mitochondrial O_2^- (mtO_2^-) from six healthy individuals. Arbitrary fluorescence units (FU) for each labeling are shown. Fluorescence means and standard deviation (SD) of basal and induced values are shown. Significant statistical differences are observed between basal and induced measurements (Paired t-test; p<0.001).

Sample	Basal mtO_2^- (FU)	Plumbagin-induced mtO_2^- (FU)
1	750	1108
2	969	2746
3	686	1847
4	853	1468
5	1345	3725
6	1190	3101
Mean	965	2499
SD	257	926

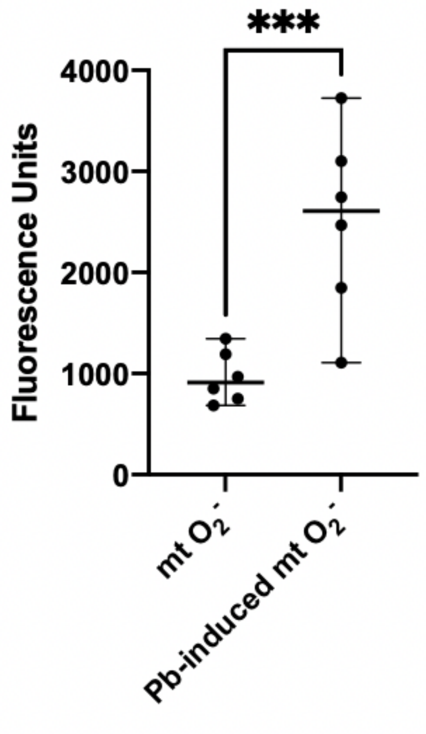

Table S11. Labeling for mitochondrial mass from six healthy individuals. Arbitrary fluorescence units (FU) for each sample are shown. Fluorescence means and standard deviation (SD) of basal values are shown.

Sample	Mitochondrial mass (FU)
1	2543
2	2176
3	2247
4	2326
5	2574
6	2722
Mean	2431
SD	213

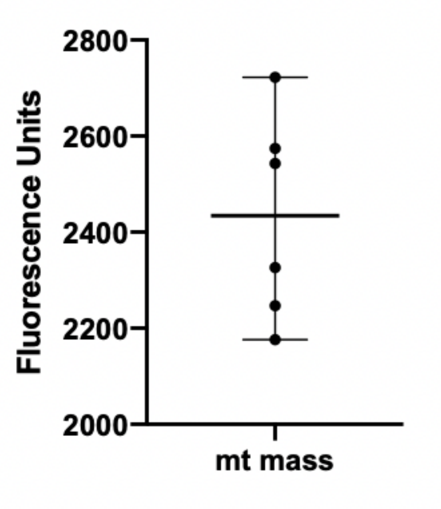

Table S12. Basal labeling and the corresponding positive control for carbonylated proteins from six healthy individuals. Arbitrary fluorescence units (FU) for each labeling are shown. Fluorescence means and standard deviation (SD) of basal and induced values are shown. Significant statistical differences are observed between basal and induced measurements (Paired t-test; p<0.001).

Sample	Basal carbonylated proteins (FU)	Menadione-induced carbonylated proteins (FU)
1	780	1504
2	844	1746
3	772	1546
4	963	1712
5	823	1675
6	741	1467
Mean	820	1608
SD	79	117

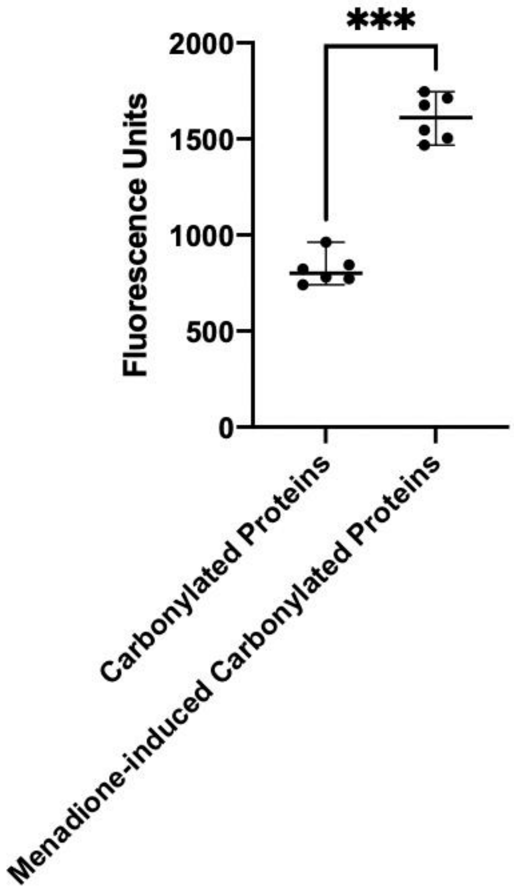

Table S13. Basal labeling and the corresponding positive control for the oxidized/reduced lipid ratio (ox/red lipid) from six healthy individuals. Arbitrary fluorescence units (FU) for each labeling are shown. Fluorescence means and standard deviation (SD) of basal and induced values are shown. Significant statistical differences are observed between basal and induced measurements (Paired t-test; p<0.001).

Sample	Basal ox/red lipid (FU)	t-BHP-induced ox/red lipid (FU)
1	147	398
2	306	1004
3	287	836
4	297	967
5	145	578
6	232	735
Mean	235	753
SD	74	233

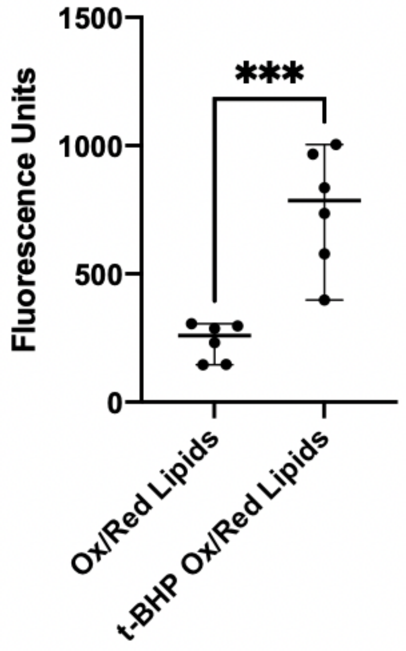

Supplement: Supplementary file 1 [file jcm-10-01172-s001.pdf]
